# The Neural Basis of Centre-Surround Interactions in Visual Motion Processing

**DOI:** 10.1371/journal.pone.0022902

**Published:** 2011-07-29

**Authors:** Christina Moutsiana, David T. Field, John P. Harris

**Affiliations:** Department of Psychology, University of Reading, Reading, United Kingdom; Rutgers University, United States of America

## Abstract

Perception of a moving visual stimulus can be suppressed or enhanced by surrounding context in adjacent parts of the visual field. We studied the neural processes underlying such contextual modulation with fMRI. We selected motion selective regions of interest (ROI) in the occipital and parietal lobes with sufficiently well defined topography to preclude direct activation by the surround. BOLD signal in the ROIs was suppressed when surround motion direction matched central stimulus direction, and increased when it was opposite. With the exception of hMT+/V5, inserting a gap between the stimulus and the surround abolished surround modulation. This dissociation between hMT+/V5 and other motion selective regions prompted us to ask whether motion perception is closely linked to processing in hMT+/V5, or reflects the net activity across all motion selective cortex. The motion aftereffect (MAE) provided a measure of motion perception, and the same stimulus configurations that were used in the fMRI experiments served as adapters. Using a linear model, we found that the MAE was predicted more accurately by the BOLD signal in hMT+/V5 than it was by the BOLD signal in other motion selective regions. However, a substantial improvement in prediction accuracy could be achieved by using the net activity across all motion selective cortex as a predictor, suggesting the overall conclusion that visual motion perception depends upon the integration of activity across different areas of visual cortex.

## Introduction

Segregating moving objects from their background and from each other is an essential step in visual processing, and the key source of information for achieving this is retinal image motion [1]–[4]. However, for the visual system to detect a moving object in the world, it is not sufficient to simply detect retinal image motion. This is because there are multiple sources of such motion, and therefore the visual system requires a method of discounting image motion that is not due to object motion. Sources of image motion that must be discounted include rotation of the eyes, movement of the head, and locomotion of the perceiver. Image motion produced by these sources occurs globally across the retina, while retinal image motion produced by motion of an object in the world tends to be localised to a part of the retina. Therefore, in a situation where the perceiver moves and an object is moving, the object can be identified as an area of the retina where local motion direction or velocity differs from that surrounding it.

Given these characteristics of retinal image motion, one low level spatial mechanism that could successfully detect object motion consists of a centre-surround organisation that compares the direction of motion in the centre and surround, with the result of the comparison being that overall activity is reduced when centre and surround motion directions are similar, and enhanced when central motion direction differs from surround motion direction. Such centre-surround spatial processing arrangements appear to exist at multiple levels of the visual system, and are not confined to the processing of motion information. Below, we briefly review evidence for centre-surround organization of the visual receptive fields of individual neurons, as well as centre-surround effects in psychophysical and brain imaging experiments that are produced by the net activity of a population of neurons whose individual receptive fields are not spatially aligned.

Nakayama & Loomis [5] suggested that detection of the kind of visual motion produced by object motion could be supported by single neurons in visual areas with centre-surround receptive field organization. Single cell recordings have subsequently shown that visual neurons often exhibit centre-surround organization [6], such that stimulation of the central classical receptive field (CRF) results in a different firing rate from simultaneous stimulation of the CRF and the surrounding region (extra CRF, ECRF). The effect of ECRF stimulation may be either to inhibit or to facilitate firing rate [6], [7].

Recent psychophysical and fMRI studies have also investigated centre-surround interactions in motion processing, effects which can be traced back at least to the work of Loomis and Nakayama [8] and these studies can be taken to reflect the net response of neural populations rather than that of individual cells. Tadin et al. [9] also showed that inhibitory centre-surround effects in motion processing depend on stimulus size and contrast, with large high contrast uniform motion producing greatest suppression, consistent with a possible role of inhibitory mechanisms in lowering activity produced by background motion. Paffen et al. [10] studied centre surround interactions in visual motion processing during binocular rivalry, in which dissimilar stimuli presented simultaneously to the two eyes compete for perceptual dominance. They showed that presenting a surround affected the dominance of rival targets in the centre. In particular, presenting surround motion increased the dominance of the centre target containing the opposite direction of motion, consistent with the proposal that centre-surround interactions in motion processing act to highlight areas of retinal motion that are more likely to correspond to object motion than motion of the background. In another study, Paffen et al. [11] showed both inhibition due to same direction of motion, and facilitation due to opposite direction of motion between centre and surround. Their stimuli consisted of two circular targets (static rings or moving gratings) that could be surrounded by an annulus composed of moving gratings. When targets were surrounded by motion in the same direction as the motion target, there was a decreased dominance of the motion in the centre (surround suppression). When both targets were surrounded by a direction of motion opposite to the motion target, the dominance of motion in the centre increased (surround facilitation).

The results of the psychophysical studies described above reflect modulation of neural activity at the population level, consistent with the existence of centre-surround organization at the population level, which might be produced by lateral connections between individual visual neurons within visual topographic maps, or by connections between multiple topographic maps. In principle these effects should be detectable using fMRI by exploiting the topographic organization of visual areas. In an fMRI study of surround modulation using static sinusoidal gratings, Williams et al. [12] found suppression of brain activation corresponding topographically to the central region of the stimulus when the surround had the same orientation as the central grating, and weak facilitation when the orientation of the surround grating was orthogonal to the central one. In a study of lateral masking, Zenger-Landolt and Heeger [13] showed that thresholds for contrast discrimination between the segments of an annulus were raised (and BOLD responses in V1 were reduced) when the annulus was surrounded by a counter-phasing grating. Apparently related suppressive effects were found in an fMRI study by Press et al [14] who showed that the activity in voxels responding to a 1.5 deg target fell in V1 as stimulus diameter was increased from 2 to 6 degrees, whereas in V3B their activity increased with stimulus size. In this study, we used fMRI to isolate cortical regions that were topographically selective for foveal motion, but which showed no activation when motion was presented in the surrounding region of visual space. We then established that the direct BOLD response produced by foveal motion could be inhibited by a simultaneously presented surround that was a smooth continuation of the central motion region, and facilitated by one that created a motion defined boundary. In a second experiment we examined whether surround effects are driven only by the region of visual space adjacent to the foveal motion, or whether longer range interactions are possible. Results indicated that longer range interactions only occur in the hMT+/V5 region, which has previously been shown to have a specific role in integrating motion signals across visual space [15], [16].

To establish that our brain imaging findings reflected actual motion perception we used the BOLD signal results to predict a perceptual effect, measured outside the scanner with a different set of participants. Prolonged viewing of an area of uniform motion produces a motion after effect (MAE), in which illusory motion is generated in the opposite direction to the motion that was stared at. Previous reports have indicated that the strength of the MAE can be influenced by the presence of a surround [17], [18], and so we measured the strength of MAEs produced by the different centre-surround stimulus configurations we used in the scanner. We reasoned that those centre-surround configurations that produced stronger BOLD signals should produce stronger MAEs when used as adapters. Given our finding that surround modulation effects differed across brain regions, we went on to ask whether surround modulation of the MAE was better predicted by the BOLD signal in hMT+/V5, or that in other extra-striate motion selective regions, or that in early visual cortex (V1/V2), or whether it reflected the net signal across different regions. Note that previous studies of the neural basis of the MAE have measured the BOLD response while participants experience the effects of motion adaptation e.g. [19]–[21] whereas our study asks a different but complementary question – is the BOLD signal a good index of the effectiveness of an adapting stimulus in producing the MAE?

## Materials and Methods

### Experiment 1: Defining ROIs and measuring surround modulation

Two sets of visual stimuli were used in this experiment, all presented to participants within the same sessions. The first set was used to define regions of interest (ROIs) in which the BOLD response to an alternately expanding and contracting circular grating presented in the central 3° of the visual field was greater than the response to a static grating. We used radial motion as an alternative to translation because, in a pilot study, translating stimuli of the necessary duration produced optokinetic nystagmaus, which prevented effective fixation. Since radial motion translates equally across all directions, fixation is more stable and it allowed us to successfully segregate the centre and surround responses. The use of radial motion was also helpful in generating a strong BOLD response to our central motion area - radially moving dots have been shown to elicit greater BOLD responses than translation at small eccentricities within the lower visual areas [22]. To prevent adaptation of the BOLD response, which we observed in a pilot study using 16 sec blocks of continuous expansion, expansion and contraction were alternated. Motion speed was 1.5 deg/sec and direction reversals occurred every 0.66 sec.

As well as the moving and static stimuli that directly stimulated the central 3° of the visual field, the first set also included circular gratings, either alternating between expansion and contraction or static, presented within an annulus, whose width was 9°, surrounding a central 3° diameter blank area. These two stimuli were used to isolate voxels where the BOLD response to surround motion was greater than that to a static surrounding grating. In the final stage of ROI definition, voxels that showed motion selectivity for both central and peripheral presentations were excluded from the ROI. The statistical threshold for the *moving centre – static centre* contrast was conservative (False Discovery Rate, *p*<0.01), while the threshold for the *moving surround – static surround* contrast was liberal (*p*<0.05, uncorrected for multiple comparisons), resulting in exclusion of voxels that had even a weak motion selectivity for surround stimulation. Thus we ensured that the ROIs contained populations of visual neurons whose topographic mapping corresponded only to the central stimulus.

**Figure 1:**
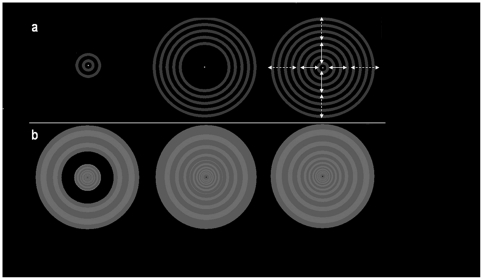
Visual stimuli. Panel a show static frames from the alternately expanding and contracting stimuli used in Experiment 1. Panel b shows the modified stimuli used in Experiment 2. Panel a (left) shows the central motion baseline, which was also used in both its moving and static form for defining the ROI. The central motion stimulus consisted of either 2 or 3 rings depending upon the stage of the expansion/contraction cycle. The static version consisted of a single frame from the moving version, chosen so that 3 rings were visible. Panel a (centre) shows the surround grating that was used in the definition of ROI for the purpose of excluding voxels exhibiting a direct response to peripheral motion. Panel a (right) shows how the two stimuli in Panel a were combined to produce a central motion area plus uniform or antiphase surround motion. The arrows indicate where the motion direction switched in the antiphase stimulus. Panel b presents examples of the “M scaled” stimuli used in Experiment 2. Panel b (left) shows the central motion area, as well as the dimensions of the gap and surround (where present). Panel b (middle) shows uniform surround motion with a thin static boundary imposed between the central 3 deg and the surround. For comparison, panel b (right) shows the uniform surround condition without the imposed boundary.

The second set of stimuli was designed to test for inhibition or facilitation of the BOLD response in the ROIs by motion in the surrounding region. One stimulus consisted of uniform expansion and contraction spanning both the central and surround regions, while in a second stimulus the surround motion was in antiphase with the central motion. We repeated the presentation of the central motion stimulus alone to provide a baseline measure of the BOLD signal change in the ROI, produced by direct stimulation of the central regions without surround modulation, which was independent of the stimuli used in ROI definition. The final stimulus was the fixation cross alone. We reasoned that suppression by a surround would result in a lower signal in the ROI than that produced by the central stimulus alone, while facilitation by a surround would produce a larger signal. The various centre/surround combinations are shown in Figure 1.

#### Visual stimulus details

The stimuli were generated using Matlab and PsychToolBox [23], [24], and back-projected on a translucent screen mounted in the bore of the scanner by a Sanyo PLC-XP40L, LCD projector (60 Hz refresh rate; 1024*768 pixels). The size of the projected image was 52.3 cm×39.0 cm. The distance from the projection screen to the mirror was 716 mm, and the distance from the centre of the mirror to the participant's eyes was 150 mm. The display subtended 35° horizontally and 30° vertically. The experiment was conducted in a dimly lit enclosure, with the only light coming from the projection screen. Stimuli were viewed binocularly, and a central fixation point (a white square of side length 0.08°) was provided. All stimuli were luminance-defined concentric rings, with 48% Michelson contrast between the brighter rings (53 cd/m^2^) and the darker background (18.2 cd/m^2^) calculated as (Lmax−Lmin)/(Lmax+Lmin). The luminance of the background when only the fixation point was shown was also 18.2 cd/m^2^. Each ring was 0.5° wide, and there were 0.5° gaps between rings, defined by the same luminance as the general background, resulting in a spatial frequency of 1 cycle/deg.

#### fMRI scanning

Stimuli were presented in a block design, in which each block lasted 16 sec. Each experimental condition was repeated seven times. The experiment was divided into three sessions, each lasting 370 sec. We chose to divide the total scan time in this way to maximise the efficiency of fixation by allowing periods for recovery and movement of the eyes at reasonable intervals.

MRI images were obtained using the standard twelve-channel head array coil of a 3-Tesla Siemens Magnetom Trio scanner. Functional volumes consisted of 36 interleaved coronal slices positioned to cover the occipital and parietal lobes (matrix 79*95, 2 mm isotropic voxels) with zero interslice gap, TR 2500 ms, TE = 30 ms, and flip angle = 90 deg. Prospective motion correction (PACE) was used to track any small head movements made by participants, allowing slices to be automatically repositioned between each TR [25]. There were three functional scanning sessions, each lasting 370 sec (148 functional volumes). At the end of the functional session, high-resolution anatomical images were acquired in 4 min 32 sec (TE = 30 ms, TR = 1960 ms, flip angle = 11, slice thickness = 3 mm, interslice gap = 0, image matrix = 256 * 256, FOV = 256 * 256 mm, voxel size 1*1*1 mm, and 176 sagittal slices).

#### fMRI data analysis

The data from each participant were processed with Statistical Parametric Mapping software (SPM2). The SPM2 motion correction algorithm was applied to identify any scans in which inter-scan motion had not been negated by PACE prospective motion correction. No such scans were identified. Volumes from each scanning session were all co-registered to the first volume of the first scan session to adjust any differences in head position or orientation between sessions. The functional series was registered to the high-resolution anatomical image before the latter image was normalized to the Montreal Neurological Institute (MNI) template. The transformation parameters computed to achieve this normalization were also applied to the functional data series. Spatial smoothing was limited to a 4 mm kernel in order to avoid blurring the boundary between direct topographic activation produced by the central stimulus and that produced by the surround. The time series was high-pass filtered using a 128 sec cut off. The activation for each participant was modelled using a linear combination of functions obtained by convolving the known temporal profile of the experimental conditions with the standard haemodynamic function of SPM plus its time derivative. As the TR was relatively short, temporal autocorrelation between volumes was a potential problem, and so the SPM2 correction for serial correlations was applied. After using contrasts to functionally define ROIs, percentage signal change caused by the experimental stimuli was extracted using the MarsBaR (MARSeille Boîte À Région d'Intérêt), [26] toolbox.

### Ethics Statement

Participants gave their informed consent in accordance with the standard procedure of the Combined Universities Brain Imaging Centre (CUBIC). All the experiments reported here were approved by the Research Ethics Committee of The University of Reading.

### Participants

Six healthy volunteers, aged 20–33, with normal or corrected vision took part in this experiment. All were familiarised with the stimuli and the demands of fixation during an initial training session on the day before they were scanned. All volunteers had previous experience in the MRI environment, and appreciated the need to remain still and fixate. They were screened according to standard procedures and written informed consent was obtained. They were paid for their time.

### Experiment 2: The effect of spatial separation between centre and surround on surround modulation

In this experiment we asked whether surround modulation of the BOLD response in ROIs defined in the same way as Experiment 1 was reduced or abolished by inserting a gap between the directly stimulated region and the surround region. In a psychophysical investigation of surround modulation of motion processing, Kim and Wilson [27] found that the effect fell off approximately linearly as the separation between the tested region and the surround increased. However, they measured the effect on the perceived direction of motion of a central stimulus induced by the direction of motion of a surrounding annulus, rather than its perceived velocity, which presumably would be the perceptual correlate of our manipulations. To assess the effect of gaps on surround modulation produced by uniform and antiphase surrounds, we reused the stimulus configurations from experiment 1, and added stimuli containing a 1.5° gap between the outer diameter of the central region and the inner diameter of the surround. This was achieved by increasing the inner diameter of the surround annulus to 6° and its outer diameter to 12°.

The second aim of Experiment 2, in conjunction with Experiment 3, was to determine the relationship between the effects of surround modulation on the BOLD response and its effects on a perceptual measure. We used the static motion after effect (MAE – the apparent motion of a stationary test field which follows prolonged inspection of a moving adaptation field) as a measure of the perceptual effects of surround modulation, reported here as Experiment 3. Pilot studies revealed that measurable modulation of the MAE by surrounds could only be obtained if the perceptual salience of the surround was increased to match that of the centre. In order to maintain direct comparability between the stimuli used to elicit BOLD responses and those used to measure perceptual effects we also increased the perceptual salience of the surrounds used in Experiment 2. In both experiments, this was achieved by M-scaling the stimuli. Previous studies of lateral interactions within visual areas have also employed stimuli that control for the cortical magnification factor in this way [27], [28]. The M-scaling procedure controls for the fall off in volume of retinotopic cortex devoted to processing each degree of visual space as distance from the fovea increases. By increasing stimulus size as a function of distance from the fovea, the amount of visual cortex stimulated can be held constant across eccentricity. The exact scaling factor required to achieve M-scaling varies between visual areas [29], as does the increase in visual receptive field size with eccentricity [30]. To achieve approximate M-scaling we picked a scaling factor of 1.35, which produced stimuli that were subjectively equally resolvable across their full radius. This contrasted with the subjective experience produced by the constant width rings used in Experiment 1, where in the outer part of the surround the direction of motion was not easily resolvable. To generate the stimuli in Experiments 2 and 3 the width and velocity of each successively more eccentric moving ring was set to be 1.35 times the width of the previous ring. The stimuli resulting from this procedure are illustrated in Figure 1b. The luminance values were 4.8 cd/m^2^ for the background, 5.27 cd/m^2^ for the darker rings, and 18.3 cd/m^2^ for the brightest rings. Therefore, the Michelson contrast defining the moving pattern was 55%.

Finally, we addressed a potential confound arising from the presence of a visible boundary between centre and surround in the antiphase surround condition of Experiment 1. The uniform surround condition did not contain a boundary, and therefore any observed differences in BOLD signal between the antiphase and uniform surround conditions might be due either to the different motion direction or the boundary. In Experiment 2, to isolate the effect of a boundary we included a version of the uniform stimulus with an imposed boundary between centre and surround. The boundary consisted of a static circle (line width 0.2°, luminance 18.3 cd/m^2^) in the same position as the boundary created by opposing motion directions in the antiphase condition.

#### fMRI

Image acquisition and analysis used the same procedures and statistical thresholds as Experiment 1. The total number of functional images was 750 per participant. Stimuli were again presented in a block design, in which each block lasted 16 sec. The experiment was divided into six sessions, each lasting 176 sec.

#### Participants

Six healthy volunteers, aged 20–33, with normal or corrected vision took part in this experiment. Three of them also took part in Experiment 1, and details of recruitment, etc, were the same as in Experiment 1.

### Experiment 3: Surround modulation of the Motion After-Effect

This experiment was conducted to permit a quantitative comparison between the effects of surround modulation on the BOLD signal and motion perception. The MAE provided a measurable index of the perception of motion. The MAE becomes stronger when the motion signal of the adapting stimulus is increased by increasing its motion coherence or contrast [31], [32]. In fMRI experiments, increasing motion strength has been associated with increased BOLD contrast (e.g. [33]) and neuronal activity [34] within the motion sensitive areas. If the strength of the MAE is a function of neural activity levels in motion selective cortex then those centre-surround stimulus configurations producing greater BOLD signals in the motion selective ROI identified in Experiment 2 should be more effective adapting stimuli, producing stronger aftereffects when the MAE is tested in the area corresponding to the central region of the adapter.

#### Visual stimulus details

To produce directional adaptation, stimuli either expanded or contracted continuously instead of alternating between expansion and contraction as in the fMRI experiments. Apart from this change the adapting stimulus spatial configurations were the same as in Experiment 2, while the MAE test region corresponded only to the central part of the stimulus – analogous to the central motion ROI in the fMRI experiments. As noted earlier, in order to equate the visual salience of the surround and the central region, we M-scaled the stimuli by increasing the thickness and speed of the rings with eccentricity.

As a comparable baseline to that used for measuring the BOLD signal in Experiment 2, we adapted observers to motion in the central 3° only. The test stimulus in all conditions was a static grating of the same dimensions as the central area. In order to increase the magnitude of the MAE, the test grating had a lower contrast than the adapting gratings [32]. The other adapting stimuli were the same as the various centre-surround configurations used Experiment 2. Each condition was performed separately for adaptation to expansion and contraction of the central region.

As in fMRI Experiment 2, three different luminance values defined the visual displays used for adaptation. The luminance values were 0.94 cd/m^2^ for the background, 9.80 cd/m^2^ for the darker rings, and 70.0 cd/m^2^ for the brighter rings, so giving a Michelson contrast for the moving pattern of 75%. The luminance of the brighter rings of the static test stimulus was 41.6 cd/m^2^, and the darker rings 31.2 cd/m^2^, resulting in a Michelson contrast of 21%. The velocity and thickness of the rings were the same as in Experiment 2. Stimuli were displayed on a PC screen with 75 Hz refresh rate and 1024*768 pixels, viewed at a distance of about 53 cm. The participant's head was supported by a chinrest and stabilized by a brow bar. The experiment was conducted in a dark room, with the only light being emitted by the PC screen.

#### Measuring the MAE

Each trial began with 40 sec of adaptation, followed by presentation of the static test stimulus. The participant was asked to judge the strength of the MAE immediately after adaptation, and indicate their judgement by assigning a number between 0 and 9, where 0 corresponded to no MAE and 9 indicated apparent motion as strong as the adapting stimulus. Estimating initial MAE velocity in this way has been shown to give a similar pattern of results over a range of conditions to that obtained when tracking the MAE produced by translating motion by moving a lever at the same velocity [35]. Each adaptation condition started 7 seconds after the MAE from the previous trial had subjectively reached zero. Each adapting condition was presented twice in total, and the order of presentation of trials was randomised between participants.

#### Participants

Twelve postgraduate students and members of staff from the University of Reading participated in the experiment. Four of them had also participated in the fMRI experiments. All participants had normal or corrected-to-normal vision, and gave their written informed consent.

#### Eye movement control experiment

Differences in the BOLD signal or MAE between conditions could potentially be explained by eye movement differences, if microsaccades or brief departures from fixation were more common for one type of stimulus than another. To test this possibility, we tracked the gaze of five participants, four of whom had participated in the fMRI experiments. Recordings were made with an SR Instruments Eyelink II (Osgoode, Ontario, Canada) at 500 Hz, with a spatial resolution of 0.1 degree. We replicated the various stimuli used in both fMRI experiments. Stimuli were presented in 16 sec blocks, as in the fMRI experiment, and the task was simply to gaze steadily at the fixation point.

## Results

### Experiment 1

#### Regions of interest

We first identified the total set of ROIs (central motion) made up of motion-sensitive voxels from occipital or parietal cortex that responded directly to central motion, but did not respond directly to surround motion. We then identified the subset of these regions that fell within hMT+/V5 (hMT+/V5 central motion). Our third set of ROIs was made up of voxels in early visual areas V1/V2 that corresponded retinotopically to the central stimulus area. Table 1 gives the stereotaxic coordinates in MNI space of the hMT+/V5 and V1/V2 ROIs. Details of how each set of ROIs was defined are given below.

**Table 1:** The mean (SD) coordinates of the functionally defined regions hMT+/V5 and V1/V2.

hMT+/V5 coordinates			V1/V2 coordinates		
x	y	z	x	y	z
**−46**(3.9)	**−74**(6.9)	**−4**(6.3)	**26**(4.5)	**−100**(2.9),	**−1**(7.6)
**49**(4.3)	**−69**(11)	**2.8**(2.4)	**−22**(2.9)	**−100**(4.3)	**−6**(9.4)
**−45**(5.8)	**−68**(11)	**−3**(4.8)	**−23**(6.5)	**−99**(6.7)	**−8**(6.7)
**46**(6.9)	**−76**(8)	**6.8**(6.2)	**29**(3)	**−98**(4.7)	**−5**(2.4)

The coordinates above and below the separation line correspond to Experiment 1 and Experiment 2 respectively.

#### Central motion ROIs

**Figure 2:**
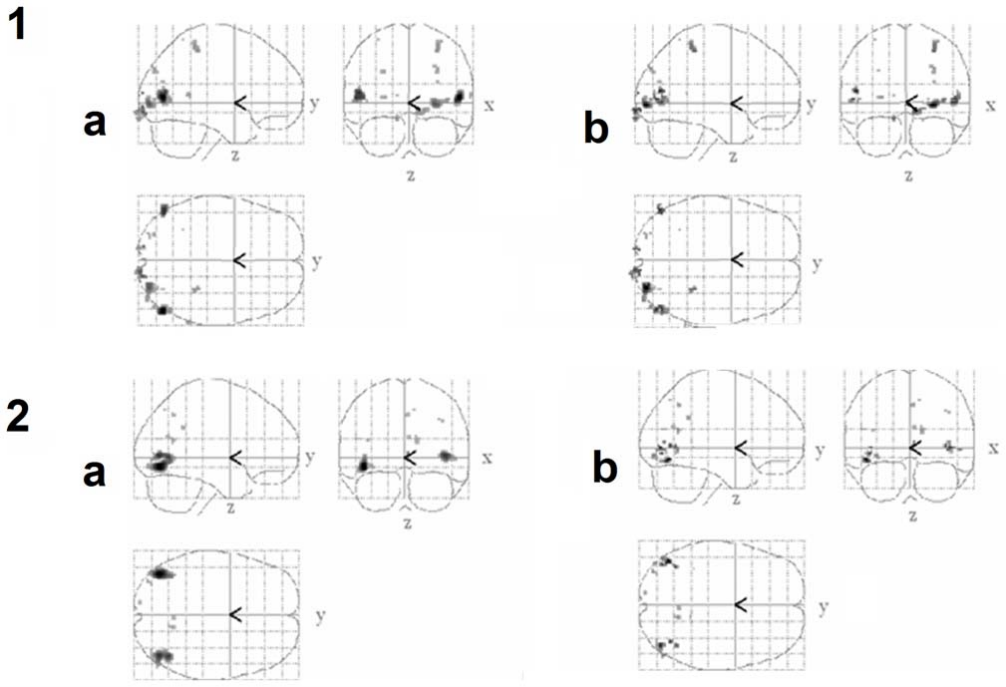
Example ROI. The two left hand panels (1a and 2a) show motion selective voxels activated by the moving centre – static centre contrast in two example participants. The two right hand panels (1b and 2b) show the subset of motion selective voxels that did not respond to motion in the surround (see Figure 1, panel a). These “central motion only” voxels were used as ROI in the Experiments. We used a liberal criterion to locate voxels that did respond to peripheral motion, thus ensuring that even voxels with a weak direct response to the surrounds we used were excluded from the ROI. See text for details of statistical thresholds.

To isolate the central motion ROIs, we first used the contrast *moving centre - static centre* (False Discovery Rate *p* < 0.01) and then excluded voxels if their activity was significant in the contrast *moving surround - static surround*, (*p* < 0.05, uncorrected for multiple comparisons). We also excluded any voxels where the initial beta weight for the moving central stimulus was negative. The *moving > static* contrast is a standard technique for identifying motion selective areas, particularly hMT+/V5 [19]. Figure 2 shows the activated voxels before and after excluding the areas directly activated by the moving surround stimuli for two example participants. In all participants, excluding the areas corresponding topographically to the moving surround stimuli decreased the number of activated voxels. Across 12 hemispheres we identified a total of 79 central motion ROIs (mean ROI volume 61 mm^3^, SE 11 mm^3^). The procedure resulted in ROIs that were in striate, extrastriate, and parietal cortex, that had some degree of retinotopic organisation and in which a moving stimulus produced a stronger signal than a static one.

#### hMT+/V5

To define the subset of the central motion ROIs corresponding to hMT+V5, we first defined hMT+/V5 in each hemisphere as the cluster with the strongest activation in the *moving centre – static centre* contrast, setting the False Discovery Rate at *p* = 0.001. This resulted in one significantly activated cluster within each hemisphere that lay close to the ascending limb of inferior temporal sulcus and lateral occipital sulcus. This is in agreement with the MT location reported in previous studies [36]. In a second step, only the ROIs from the central motion set that lay within that cluster were included in the hMT+/V5 central motion set (N = 28). ROIs in the hMT+/V5 were available for 10 out of 12 hemispheres (mean ROI volume 52 mm^3^, SE 14 mm^3^).

#### V1/V2

We used the fact that early visual cortex has a strong response for static visual stimuli and fine retinotopic organisation to produce an independent localizer for that region. V1/V2 ROIs were defined by the contrast *static central - rest* (False Discovery Rate *p*<0.001), with voxels activated even weakly (*p*<0.05 uncorrected) by the static surround excluded. This produced a cluster in the calcarine fissure which combined the central visual field representations of V1 and V2. For each hemisphere only the region with the peak activation, which was also the cluster with the most voxels in/around the calcarine fissure was included in the analysis (N = 12, mean ROI volume 1545 mm^3^, SE 559 mm^3^).

#### Surround Modulation

**Figure 3:**
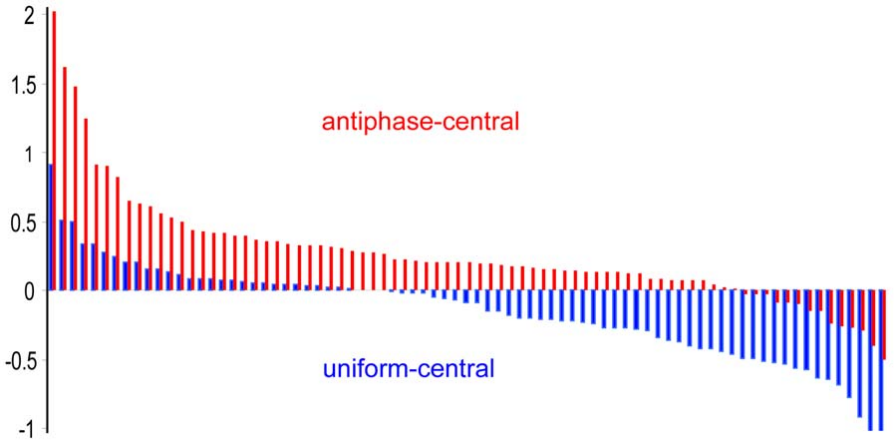
Experiment 1: The effects of surround modulation on percent BOLD signal change in individual motion selective ROI. For each of the 79 central motion ROI the difference in signal change between the uniform motion surround condition and the central motion baseline is plotted in blue; suppression is reflected in negative y axis values. The ROI have been sorted to group together those where the surround caused most suppression on the right. A smaller number where it had a negligible effect or caused facilitation are grouped on the left. For the same ROI the red bars show the difference in signal change between the antiphase surround and the central motion baseline. The red data bars have been sorted to show those ROI where the surround caused the strongest facilitation on the left.

We calculated the percentage BOLD signal change in each of the central motion ROIs under the three experimental conditions (central motion, uniform motion across centre and surround, and antiphase surround motion). Figure 3 presents a visual comparison, in which, for each central motion ROI, the percentage signal change in the central motion baseline condition has been subtracted from that in each of the surround conditions. For nearly all ROIs, the difference is positive when the surround moves in antiphase to the centre, indicating facilitation. For over half the ROIs, the difference is negative when the surround moves in the same direction, indicating suppression. This pattern of results was found in both the hMT+/V5 central motion ROIs and the V1/V2 ROIs.

**Figure 4:**
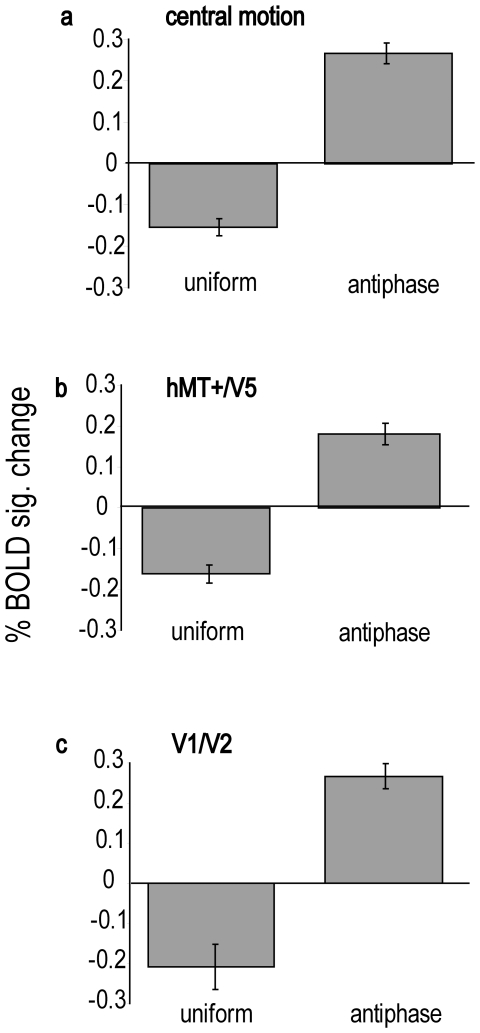
Experiment 1: the net effect of surround modulation on BOLD signal change in each of the three sets of ROI. Panel a shows signal change averaged across the full set of 79 central motion ROI when surround motion was either in phase (uniform) with central motion or in antiphase to it. Signal change is plotted relative to the signal change obtained in the central motion baseline, which is itself represented by the value 0 on the y axis. Panel b makes the same comparison for the 28 hMT+/V5 central motion ROI, and panel c shows the effect of surround modulation in the 12 V1/V2 ROI. Error bars indicate one standard error.

The mean signal change in the set of central motion ROIs for the uniform motion surround and the antiphase surround relative to the central motion baseline is shown in Figure 4a. Same direction motion in the surround significantly decreased the percentage signal change compared to the central motion only baseline, t (78) = 3.89, *p *< 0.001. The effect size of the difference was 0.43 (Cohen's d, the mean difference divided by the standard deviation of the difference scores). Antiphase motion in the surround significantly increased the percentage signal change compared to the central motion only baseline, t (78) = 5.75, *p *< 0.001, with Cohen's d = 0.65.

The mean signal changes in the set of hMT+/V5 central motion ROIs for the uniform motion surround and the antiphase surround relative to the central motion baseline are shown in Figure 4b. Uniform motion across centre and surround significantly decreased the percentage signal change compared to the central motion baseline, t (27) = 4.31 *p *< 0.001, and d = 0.81. Antiphase motion in the surround significantly increased the percentage signal change compared to the central motion baseline, t (27) = 4.05, *p *< 0.001, d = 0.77. The effect sizes were larger in the hMT+/V5 central motion ROIs than in the full set of motion selective regions due to relatively lower variation between regions in hMT+/V5.

The mean signal changes in the set of V1/V2 ROIs for the uniform motion surround and the antiphase surround relative to the central motion baseline are shown in Figure 4c. Same direction motion in the surround significantly decreased the percentage signal change compared to the central motion baseline, t (11) = 2.29 *p *< 0.05, d = 0.66. Antiphase motion in the surround significantly increased the percentage signal change compared to the central motion baseline, t (11) = 6.36, *p *< 0.001, d = 1.83. The very large effect size for the antiphase versus baseline comparison was due to the combination of a relatively large mean difference and relatively low variation between regions.

### Experiment 2

#### Regions of interest

ROIs were defined using the same procedures as Experiment 1, the only difference being that the stimuli were M-scaled (see Methods). In 12 hemispheres, we identified 64 central motion ROIs (mean ROI volume 73 mm^3^, SE 27 mm^3^), 14 of which were hMT+/V5 ROIs (mean ROI volume 51 mm^3^, SE 31 mm^3^), and 12 V1/V2 ROIs (mean ROI volume 1564 mm^3^, SE 422 mm^3^) that were selective for the central 3 degrees of the visual field, but not specifically motion selective. In order to permit necessary statistical comparisons, we also defined a fourth category of ROI, consisting of the 50 motion selective areas that were not included in the hMT+/V5 subcategory, which we refer to as central motion not MT+/V5 ROIs (mean ROI volume 87 mm^3^, SE 33 mm^3^). The stereotaxic coordinates in MNI space of the hMT+/V5 and V1/V2 ROIs are given in Table 1.

#### Surround Modulation

**Figure 5:**
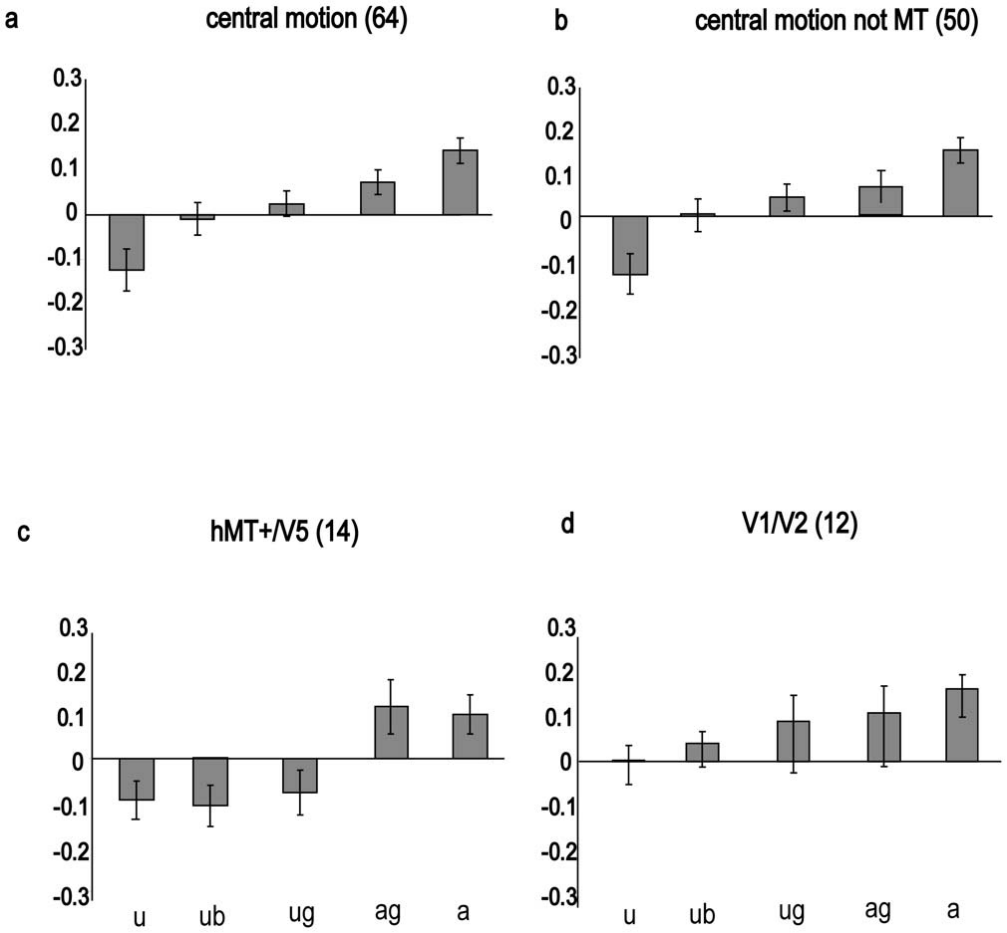
Experiment 2: the net effects of gaps and boundaries on surround modulation of the BOLD signal in each of the four sets of ROI. Panel a y axis plots percent signal change in surround conditions averaged across the full set of 64 central motion ROI relative to the percent signal change obtained in the central motion only baseline, which is itself represented by the value 0 on the y axis. From left to right, bars indicate signal change when surround motion was in phase with central motion (u); surround was in phase but a static boundary was imposed to mimic the motion defined boundary present in the antiphase condition (ub); surround was in phase and separated from the centre by a gap (ug); surround was antiphase and separated from the centre by a gap (ag); or surround was antiphase and continuous with the central region (a). Panel b presents the signal change in the 50 motion selective ROI that lay outside hMT+/V5. Panel c presents the signal change in the 14 hMT+/V5 central motion ROI, and panel d shows the effect of surround modulation in the 12 V1/V2 ROI. Error bars indicate one standard error.

We calculated the percentage BOLD signal change in each ROI for the five experimental conditions and the central motion baseline. In Figure 5 the BOLD signal in each experimental condition relative to the central motion baseline is shown separately for each of the four categories of ROI. While the three categories of ROI that were defined on the basis of motion selectivity showed the same basic surround modulation effects revealed in Experiment 1, the effect of inserting a gap between centre and surround, as well as the effect of imposing a static boundary in the uniform motion condition, differed across ROI category. The V1/V2 ROIs showed facilitation when the surround moved in antiphase to the central motion area, but with M-scaled stimuli suppression by a surround moving in the same direction as the central region was not evident.

Considering first the full set of 64 motion selective regions, only the uniform and antiphase conditions resulted in a BOLD signal that was significantly different from the central motion baseline, uniform t(63) = −2.03, *p *< 0.05, d = −0.25, antiphase t(63) = 2.60, *p *< 0.05, d = 0.32. Conditions with imposed static boundaries or gaps produced a BOLD signal that was not significantly different from baseline, consistent with abolition of surround modulation. Further statistical evidence that surround modulation was abolished by gaps is provided by the direct comparison between antiphase surround motion with and without a gap, which was significant, t(63) = −2.22, *p *< 0.05, d = −0.28, as was the comparison between uniform surround motion with and without a gap, t(63) = 2.40, *p *< 0.05, d = 0.30. Imposing a static boundary between centre and surround in the uniform motion condition abolished suppression relative to the central motion baseline, whereas the increase in the BOLD signal relative to uniform motion without a boundary approached significance, t(63) = −1.62, *p* = 0.11, d = 0.20. While the presence of a boundary acted to prevent suppression, inspection of Figure 5a shows that it did not by itself produce facilitation comparable to that produced by antiphase motion, and therefore the boundary produced by antiphase surround motion here and in Experiment 1 can be ruled out as an explanation of facilitation.

The 50 motion selective regions (see Figure 5b) that were not identified as hMT+/V5 revealed a pattern of surround modulation that was similar to the full set. The antiphase condition produced a BOLD signal that was higher than the baseline, t(49) = 2.21, *p*<0.05, d = 0.31, while there was a trend towards significance for the reduced BOLD signal in the uniform motion condition, t(49) = −1.87, *p* = 0.067, d = 0.26. None of the conditions with gaps or boundaries produced a BOLD signal that was significantly different from baseline. Further statistical evidence that surround modulation was reduced by gaps is provided by the direct comparison between antiphase surround motion with and without a gap, which was significant, t(49) = 3.04, *p* < 0.01, d = 0.42 as was the comparison between uniform surround motion with and without a gap, t(49) = 2.45, *p* < 0.05, d = 0.34.

Turning to the subset of motion selective regions that were identified as hMT+/V5 central motion ROIs, the pattern of results is qualitatively different (see Figure 5c) from that in the other motion selective regions. In these regions, surround modulation was not reduced or abolished by the introduction of a gap or boundary between centre and surround. As can be seen from the error bars, the comparison of any uniform motion surround with any antiphase surround was statistically reliable. Perhaps the most striking result was that when the surround was separated from the central area by a gap, reversing the direction of motion in the surround was sufficient to produce a clear difference in activation, t(13) = 2.55, *p* < 0.05, d = 0.68. These results are in agreement with the role of MT in integrating motion signals across visual space.

To confirm that the effect of the boundary and the gaps on BOLD responses in hMT+/V5 was different from their effects in the other motion selective ROI we made direct comparisons between the two categories of ROI using independent samples t tests. Levene's test indicated that the variance of the two ROI categories was significantly different in all the following comparisons, and therefore t statistics and degrees of freedom have been adjusted to take this into account. As suggested by Figure 5 (panels a and b), there was no significant difference between the two ROI categories in either the uniform or antiphase conditions. There was a significantly lower BOLD response in hMT+/V5 to both the uniform boundary condition, t(49.19) = 2.07, *p* < 0.05, d = 0.53 and the uniform gap condition, t(53.07) = 2.19, *p* < 0.05, d = 0.55. However, there was no significant difference in BOLD response to the antiphase gap condition, t(42.48) = 1.331, *NS*, d = 0.46.

In the V1/V2 ROIs, only antiphase surround motion produced strong and significant surround modulation when compared to the central motion baseline, t(11) = 4.81, *p* < 0.001, d = 1.39. This condition also produced higher signal change than the uniform surround, t(11) = 3.33, *p* < 0.01, d = 0.96, and the uniform surround with an imposed boundary, t(11) = 2.59, *p* < 0.05, d = 0.74. In contrast to the comparable condition of Experiment 1, uniform surround motion did not produce suppression relative to the baseline.

### Experiment 3

#### Motion after effect

Experiment 2 revealed that introducing a 1.5° gap between centre and surround generally removed surround modulation effects on the BOLD signal, with the notable exception of ROIs located within hMT+/V5, where surround modulation persisted despite the gap. The fMRI results suggested two possible effects of the gap in the MAE experiment. If the MAE depends primarily on processing in the specialized motion processing region hMT+/V5 then surround modulation of the MAE would not be influenced by the gap. However, if the MAE depends upon the net firing rates across motion selective areas of cortex, then the gap would abolish surround modulation of the MAE.

**Figure 6:**
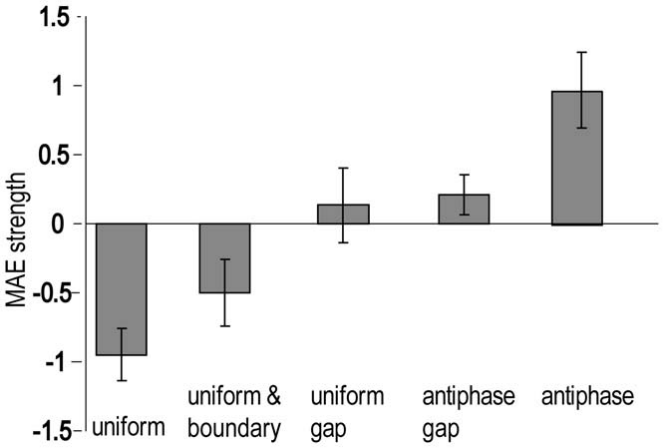
Experiment 3: the effects of gaps and boundaries on surround modulation of the MAE. Mean strength ratings are shown relative to the central motion baseline, which is represented by the value 0 on the y axis. From left to right, in the same order as Figure 5, bars indicate signal change when surround motion was in phase with central motion (uniform); surround was in phase but a static boundary was imposed to mimic the motion defined boundary present in the antiphase condition; surround was in phase and separated from the centre by a gap; surround was antiphase and separated from the centre by a gap; or surround was antiphase and continuous with the central region. Error bars indicate one standard error.

MAEs were stronger when the direction of motion in the central region of the adapting stimulus was contracting [37], but this effect did not interact with any other effect, and so the results presented are collapsed across the two adaptation directions. The mean MAE strength ratings are presented in Figure 6, in which each condition is shown relative to the central motion baseline, following the same format as the BOLD percent signal changes in Figure 5.

The pattern of MAE results was strikingly similar to the pattern of BOLD signal changes obtained for the full set of central motion ROIs in fMRI Experiment 2. This suggests that the subjective MAE reflects the net activity across all motion selective areas of visual cortex rather than hMT+/V5 specifically, because hMT+/V5 had a different pattern of BOLD responses to the centre-surround stimulus configurations we used (Figure 5c). Statistically, the MAE results were also similar to those of the central motion ROIs in Experiment 2. Only the uniform and antiphase conditions resulted in a MAE that was significantly different from the central motion baseline, uniform t(11) = −2.93, *p* < 0.05, d = −0.84, antiphase t(11) = 2.34, *p* < 0.05, d = 0.67. Conditions with imposed static boundaries or gaps produced an MAE that was not significantly different from baseline, consistent with abolition of surround modulation. Further statistical evidence that surround modulation was abolished by gaps is provided by the direct comparison between uniform surround motion with and without a gap, which was significant, t(11) = −2.67, *p* < 0.05, d = −0.77, as was the comparison between antiphase surround motion with and without a gap, t(11) = 2.44, *p* < 0.05, d = 0.71. Imposing a static boundary between centre and surround in the uniform motion condition abolished suppression relative to the central motion baseline, but the increase in the MAE relative to uniform motion without a boundary only approached significance, t(11) = 2.12, *p* = 0.058, d = 0.61.

#### Using the BOLD signal to predict the MAE

**Figure 7:**
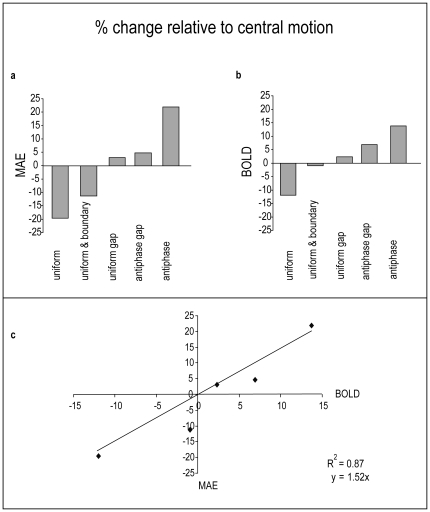
Relationship between BOLD signal change and MAE strength. To facilitate a visual comparison between the results of Experiments 2 (BOLD) and 3 (MAE) panels a and b shows the differences from the central motion baseline caused by each surround condition converted to percentages of the respective baselines. The BOLD data in panel a is the mean of the set of 64 central motion ROI, which predicted the MAE more accurately than the data from the other ROI categories. The MAE shows greater change as a percentage of its baseline than the BOLD signal, but a linear coupling between the two measures is apparent. Panel c shows the MAE as a function of the BOLD signal in the equivalent condition, indicating that a simple linear model constrained to assume that when BOLD is equal to its baseline MAE should also be at baseline is able to account for 87% of the variance in the MAE, even though the MAE and BOLD were measured in two different groups of participants.

Although the MAE and fMRI experiments were conducted with different groups of participants, and the MAE is an indirect measure of centre-surround interaction, the pattern of results for the MAE was strikingly similar to the BOLD signal averaged across the full set of motion selective regions that we identified. We made the assumption that the BOLD signal, as a proxy for neural activity during adaptation, predicted the MAE, rather than the other way around. To render the BOLD data from the four different ROI categories and the MAE data directly comparable, we converted both sets of data to the percentage change from their respective central motion baselines. Thus, the change in the BOLD signal relative to baseline in each of the five surround conditions was converted to a percentage of the raw percent signal change observed in the baseline itself (1.04 for the central motion ROIs, 0.72 for hMT+/V5, 1.1 for motion selective ROIs that were not hMT+/V5, and 1.82 for the V1/V2 ROIs). For the MAE, the differences were converted to a percentage of the mean strength rating received by the baseline (4.42 on the 0–9 scale employed). An example of this, illustrating the good correspondence between the MAE and BOLD in the central motion ROIs can be seen in Figure 7a and 7b.

To provide a quantitative comparison, for each ROI category in turn, linear regression was used to predict the MAE from the BOLD signal. For regression lines to describe a meaningful relationship between BOLD and the MAE the fits had to conform to an underlying assumption that when the BOLD signal (as a proxy for neural activity) was at its baseline level, the MAE should also be at its baseline level. Therefore, we required the fitted regression lines to pass through the origin. A further assumption required for BOLD to meaningfully predict the MAE was that when BOLD was below baseline, the MAE should also be below baseline, and when the BOLD signal was above baseline the MAE should also be above baseline. This constrained us to exclude any fitted regression lines with negative slopes (there were none).

The best fit was obtained using the BOLD signal from the full set of 64 motion selective ROIs as the predictor. This is shown in Figure 7c, which shows the MAE as a function of BOLD in the equivalent experimental condition. The experimental conditions are shown in the same order from left to right as in Figures 7a and 7b. The fit for the central motion ROIs was better than that obtained using any of the other ROI categories, including hMT+/V5, but this depended to some extent on the larger number of ROIs contributing to the prediction, and included the contribution from hMT+/V5. To make a valid comparison between the fit obtained with the 14 hMT+/V5 ROIs and the fit that could be obtained using the BOLD signal from other motion selective regions required the number of ROIs in each category to be matched. Because there were 50 motion selective ROIs not identified as hMT+/V5, we fitted the regression model using a random subsample of 14 of them. To control for the dependency of the obtained fit on the specific regions selected we repeated the subsampling procedure 10000 times and calculated the mean fit. The results of this procedure showed that hMT+/V5 provided a substantially better fit to the MAE data than the other motion selective regions.

While the subsampling procedure controlled for the influence of the number of ROIs on the model fit, the hMT+/V5 ROIs were on average smaller than the other motion selective regions, and exhibited less variability in size (see above). Therefore, each subsample of 14 regions typically contained a larger number of voxels than the hMT+/V5 set, and the greater signal averaging may have resulted in a less noisy BOLD signal than that obtained from hMT+/V5. If this was the case then it is possible that our analysis overestimated the predictive power of the non-hMT+/V5 motion-selective cortex relative to the hMT+/V5 regions. We checked for this possibility by asking whether variation in the total number of voxels in each random subsample of 14 regions covaried with the obtained fit to the MAE data. If the fit improved as the number of voxels increased, then this would indicate an important role of the number of voxels in determining the fit that was independent of the role of number of ROIs. In practice, this possibility was ruled out because there was no systematic linear or nonlinear relationship between total number of voxels used to predict the MAE and the R^2^ quantifying the strength of the relationship between BOLD and MAE. Across the 10000 subsamples both the R^2^ values and the number of voxels showed substantial variation, the mean R^2^ being 0.45 (SD 0.24) and the mean number of voxels in a set of 14 regions being 152 (SD 94), but almost none of this variation was shared (Pearson's *r* = 0.029).

The regression analyses for all the ROI categories are shown in Table 2. The best prediction was obtained using the full set of motion selective ROIs (also shown in Figure 7), followed by the subset that did not include hMT+/V5. Controlling for the number of regions, as described above, the best fit was provided by hMT+/V5. It is notable that the fit to the MAE data provided by the BOLD signal in the V1/V2 ROIs was substantially worse than the fit provided by any of the motion selective ROI categories. This result holds when the number of ROIs is similar in all three regions (14 in hMT+/V5, 14 in non-hMT+/V5 by subsampling, 12 in V1/V2).

**Table 2:** Linear regressions describing the relationship between the BOLD signal in each ROI category and MAE strength.

ROI category(N)	Slope	*R* ^2^	*p* value
All Motion (64)	1.52	0.87	**0.004**
Central motion not hMT+ (50)	1.53	0.84	**0.005**
hMT+ (14)	0.78	0.60	**0.035**
*Central motion not hMT+ (14)*	*0.84*	*0.45*	*0.105*
V1/V2 (12)	0.54	0.28	0.138

The values in italics for *Central motion not MT (14)* are the mean of 10000 sub-samples of N = 14 from the initial set of 50. We predicted positive slopes and therefore report one tailed p values – significant regressions highlighted in bold.

#### Eye movement control experiment

**Figure 8:**
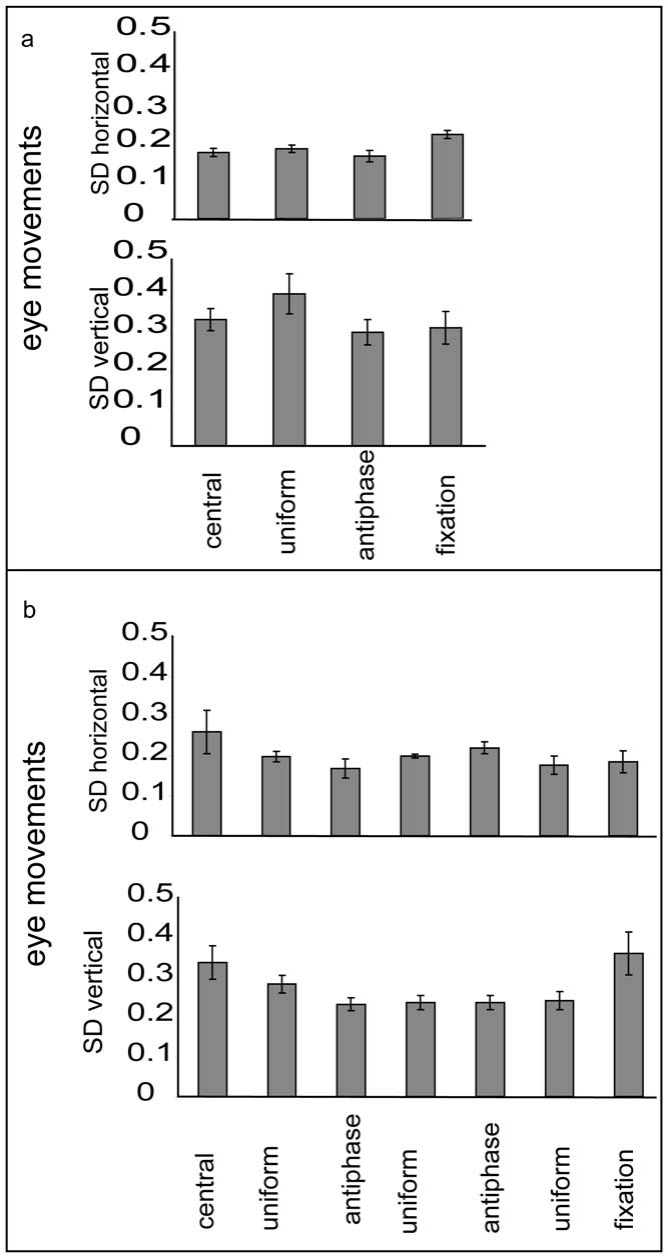
Eye movement control experiment. If the efficiency of fixation differed between experimental conditions, and the pattern of differences was similar to the differences in the BOLD signal and MAE between conditions, then eye movements could form the basis of an alternative explanation of our findings. We quantified the efficiency of fixation using the standard deviation of eye position in the horizontal and vertical directions. Panel a presents the eye movement data for the visual stimuli used in Experiment 1 (“fixation point” is equivalent to the resting baseline). Panel b shows the same data for the stimuli used in Experiments 2 and 3. The efficiency of fixation was similar across conditions, and there was no systematic pattern to suggest eye movements played a role in our findings. Error bars indicate one standard error.

If the extent of small deviations from fixation differed between experimental conditions, and the pattern of differences was similar to the differences in the BOLD signal and MAE between conditions, then eye movements might potentially form the basis of an alternative explanation for our findings. However, this is not borne out in the eye movement data presented in Figure 8. While the BOLD signal and the MAE strength showed a linear relationship, there is no obvious relationship between the results of Experiments 1, 2, and 3, and the pattern of eye movements in Figure 8. The only statistically significant comparison in the eye movement data is that between gazing at a fixation point and the antiphase condition from Experiment 1. The eyes moved more in the horizontal direction for the fixation point condition than in the antiphase condition, t(4) = 2.84, *p* < 0.05, d = 1.27.

## Discussion

### Summary of Results

We located regions of the occipital and parietal lobes that responded to moving stimuli presented in the central visual field, and which were not activated by visual motion occurring at eccentricities of 1.5 deg or greater. BOLD responses in these regions were suppressed if a surround moved in the same direction, so that central visual stimulation became part of a large field of uniform motion. On the other hand, when the surrounding motion generated a motion-defined boundary with the central region, BOLD responses were facilitated. This mechanism potentially enables motion selective cortex to distinguish which of two otherwise identical local motion signals arise from background motion and which arises from object motion. In the context of the figure-ground segregation problem, the ability to integrate signals across a greater range, which we only found evidence for in hMT+/V5, may have a role in allowing regions of visual space to be grouped and classified on the basis of their direction of motion despite intervening areas of occlusion produced by objects closer than the depth plane of interest.

Our other main finding was that the strength of MAE produced by adapting stimuli with the same center-surround configurations as those used in the fMRI experiment was most accurately described by a linear function of the BOLD signal averaged across all the motion selective cortex identified in the fMRI experiment. The most likely explanation of this linear result is that there is an approximately linear relationship between the BOLD signal and neural activity as often assumed by fMRI analysis methods [38], and that there is also a linear relationship between the strength of neural activity in motion selective cortex during adaptation and the initial strength of subsequent MAEs.

### Relationship to previous psychophysical studies

Previous psychophysical studies of contextual modulation using both moving and static stimuli have found that the effects fall off as the spatial separation between the main stimulus and the context increases [27], [39]. Some investigations of surround modulation of the MAE have found that the MAE is suppressed by surround motion in the same direction as that in the test region, and enhanced when surround motion is in the opposite direction [17], while other studies find only suppression [4]. We found both effects, although our experiment differed from [17] in that we used a foveal rather than a peripheral test presentation, used radial expansion/contraction rather than translating gratings, and also investigated the effect of a gap on surround modulation. Our main motivation for carrying out the MAE experiment was to explore the relationship between a perceptual phenomenon and BOLD activity. We found that, with moving stimuli, contextual modulation of the MAE was abolished by a 1.5 degree separation between the context and the main stimulus. Furthermore, suppression of the MAE caused by a surrounding context was reduced if a static boundary was imposed between the central and surrounding regions. In terms of BOLD responses, introducing a gap or an imposed static boundary tended to abolish surround modulation in the group of ROI that were outside hMT+/V5. Within hMT+/V5 itself a different pattern emerged, in which centre-surround interactions remained as strong with the gap or imposed boundary as without them. This region is known to integrate motion signals across visual space [15], [16], and has foveal CRFs that are large enough to encompass the central region of our stimuli [40]. Given the size of the CRFs in hMT+/V5, it is likely that the ECRFs were large enough to span the 1.5 degree gap we used, as well as the stimulus surround [41]. Recently, Tadin et al [42] showed that duration thresholds for detecting the direction of large (16 deg diameter) but not small (2.4 deg diameter) drifting gratings could be improved by disrupting hMT+/V5 with TMS. They explained the improved performance by suggesting that TMS prevented surround suppression in hMT+/V5, a mechanism consistent with our MAE and BOLD data. Interestingly, when TMS was applied to V1/V2, no significant effect of surround suppression was found. Our failure to find evidence for this in Experiment 2 suggests that the conditions for eliciting it may be more critical than for hMT+/V5.

### Which brain regions best predict MAE strength?

Given the emphasis on hMT+/V5 as the main centre of motion processing, it was a reasonable expectation that the effects of contextual modulation on a measure of motion perception would be predicted by the BOLD effects in hMT+/V5, rather than elsewhere in the brain. We did find that hMT+/V5 provided a better fit to the MAE than other motion selective regions if the number of other motion selective ROIs contributing to the fit was limited to be the same as the number of hMT+/V5 ROI. The fit provided by hMT+/V5 was also considerably better than that from the V1/V2 ROIs. This is consistent with the established view that hMT+/V5 is the main centre of motion processing. However, our findings also emphasize the role of other motion selective regions in generating the conscious experience of motion. When the averaged signal from the other motion selective regions was used to predict the MAE the achieved fit was actually higher than that for hMT+/V5 alone, and the best fit was achieved by averaging the signal from hMT+/V5 and the other regions. The involvement of centre-surround interactions in regions other than hMT+/V5 in generating the MAE has been also suggested by studies on surround interactions during binocular rivalry [11], [12]. Overall our findings and those of the rivalry studies suggest that the MAE reflects the net activity of multiple cortical regions rather than solely the activity in a particular region such as hMT+/V5. Our findings also suggest the hypothesis that the magnitude of perceptual after-effects in general may be predictable from the BOLD activity in relevant areas of cortex during adaptation.

### Relationship to previous physiological studies

Previous investigations of surround modulation in human visual cortex have used static stimuli, and have focused on early retinotopic visual areas [12], [43], [44]. The results in our primary-visual ROIs with moving surrounds are generally consistent with previous results obtained using static stimuli. Experiment 1, using unscaled stimuli, produced results similar to those of Williams et al. [12], except that in our case facilitation was the stronger of the two effects, while in their case surround suppression was stronger. Experiment 2 also produced facilitation due to surrounding context in V1/V2, although suppression did not occur with the M-scaled stimulus configurations. There is one previous investigation of surround modulation used fMRI and moving stimuli [45]. However they manipulated coherent versus incoherent motion and their analytic approach relied on whole brain contrasts rather than topography and ROI analysis as used here, so the two sets of findings should be compared with caution. Nonetheless, their finding of reduced activation in hMT+/V5 and primary visual cortex, when motion is coherent relative to incoherent, is consistent with our finding that uniform surround motion produces a reduced signal compared to antiphase motion. The facilitation found in V1/V2 with anti-phase stimuli may appear surprising, given the conditions under which this typically occurs in single cells in V1, namely when the cell is only weakly excited, as by a small or low contrast stimulus (see Angelucci and Bressloff, [46] for review). The apparent discrepancy may have arisen because stimuli such as ours have not been presented to motion sensitive cells in V1, and/or because, at the population level, cell-cell interactions produce a different pattern of activity from that within one receptive field. Another apparent discrepancy between our data and the properties of single cells comes from consideration of the sizes of receptive fields in human visual cortex, which are likely to be less than 1 degree in the foveal regions of V1 and V2 [47]. In our study, and that of Williams et al, the central stimulus was 3 deg in diameter, and so might be thought to suppress activity in such cells. Yet, in both studies further suppression as well as facilitation could be demonstrated. This might arise because, as already suggested, the properties of cell populations differ from those of individual cells, or because the outer regions of our central stimulus would be falling on only parts of receptive fields, and so the rest of the receptive field would be available to mediate suppression or facilitation by appropriate stimuli.

### Possible roles of attention

BOLD responses in visual cortex can be modulated by attention [20]. How do the findings reported here relate to the spatial deployment of attention? Spatial attention can be directed by top-down intentions of the observer, or captured bottom-up, by stimulus driven saliency. Considering stimulus driven attention first, the obvious low level differences between the different stimuli we used may have driven bottom-up attention shifts, especially at the onset of each 16 sec stimulus period. In fact, it has been proposed that the mechanisms underlying the stimulus saliency based deployment of attention are of the centre-surround type [48]. Therefore, one view of how the population level neural mechanisms we reveal relate to attention is that they reveal one of the mechanisms of attentional deployment at work.

Turning to the top-down deployment of attention, we think it is unlikely that this process influenced our data. Participants were given the task of maintaining central fixation, and the eye tracking experiment indicated that they were largely successful in this. Nonetheless, it might be argued that attention was shifted (or distracted) covertly towards surrounds when they were present and formed some kind of boundary with the central region of the stimulus, and that such shifts accounted for variation in the BOLD response produced by the central stimulus. However, if participants did shift spatial attention towards surrounds in the gap conditions of Experiment 2, then the BOLD response to the central stimulus should have declined relative to the central stimulus only baseline. This should have happened regardless of whether surround motion was in or out of phase with motion in the central region. Contradicting this account, the BOLD response to the central region was simply uninfluenced by a surround if there was a gap, except in hMT+/V5, where there was an effect, but not of the unidirectional type predicted by this attentional distraction account. Furthermore, the attentional distraction account does not predict the different pattern of results we found in hMT+/V5 compared to other motion sensitive ROIs. Therefore, our results are more likely to reflect the low level mechanisms of stimulus driven saliency than top-down attentional selection.

### Possible role of eye movements

Our paradigm relies upon the topographic organisation of visual areas, as well as the ability to present visual stimuli at a consistent retinal location. The concentric pattern of expansion and contraction we used was specifically designed to remove any visual signal that would produce optokinetic nystagmus (OKN). Although attempting to fixate, participants inadvertently make small eye movements, and if these were more prevalent in some conditions our findings might be confounded by BOLD activation produced by making eye movements, or by changes in the topographic location of visual stimulation that eye movements cause. However, analysis of eye movements showed that this explanation of the findings is highly unlikely - ocular behaviour was similar in all the experimental conditions we tested. The small differences between conditions that did occur showed no consistent relationship with the experimental conditions, unlike BOLD and the MAE.

### Topographic analyses

Although our paradigm relied upon the well documented topography of visual areas to define ROIs, we did not acquire full retinotopic maps of our participants' visual systems. Such maps would have been of limited use in locating our functionally defined ROIs within specific visual areas because our test stimuli were foveal, and conventional retinotopic mapping possesses insufficient spatial resolution to separate the different visual areas within the central 2 deg of visual space. Given that we measured signal change in ROIs corresponding to the central 3 deg of visual space, retinotopic mapping data would, at best, have, allowed us to separate V3A/B from V1/V2, which would not have influenced our main conclusions. In fact, our ROI definition procedure was a simple form of visual eccentricity mapping because voxels were only included in a ROI if they responded to stimulation of the central 3 degrees of visual space, but did not respond to more eccentric visual stimulation. Centering our centre-surround stimulus at several degrees of eccentricity instead of at the fovea would potentially have allowed us to take advantage of information provided by retinotopic mapping. This was attempted in a pilot study, but due to the much smaller volume of cortex that represents peripheral visual space we were unable to locate voxels that responded to the centre of the stimulus but not the surround, which was a necessary criterion for testing our hypotheses.
